# 1, Case of Gun-shot Wound in the Chest—2, Poisoning by Laudanum

**Published:** 1860-07

**Authors:** A. Groesbeck

**Affiliations:** Chicago, Ill.


### ARTICLE 7.

### 1. CASE OF GUN-SHOT WOUND IN THE CHEST.—2. POISONING BY LAUDANUM.

BY A. GROESBECK, M. D., CHICAGO, ILL.

May 5.—Was called up at 12 o'clock at night, to see a man who had been shot with a pistol ball. Found a small round wound in the center of the sterum about midway of its length. There was very little hemorrhage externally, and no expectoration of blood or disposition to cough. The pulse small and feeble and the extremities cold. On moving him, blood and air escaped from the wound. By auscultation the left lung was discovered to be wounded, and the bubbling of air in effused fluid was heard. I gave him opium and brandy freely, and at 4 A. M., a good reaction took place.

May 6.—Restless and complaining of pain in the chest. I continued the use of one-half gr. of morphia every three hours during the day.

May 7. Very comfortable; pulse 120, not very full; morphia given when required by pain. On percussion, left side very resonant, indicating effusion of air; bubbling sound in left side continues; respiration of right side natural.

May 8.—Patient free from pain; sleeps well; discontinue morphia.

May 9 and 10.—Condition the same except expectorating a few sputa containing blood, which ceased from that time, neither has there been any expectoration since, of any kind. Respiration in the right lung continued natural until the 13th, when the whole lung appeared to be solid. No respiration could be heard throughout the whole right side. This continued for several days, when respiration began to be heard at the top of the lung, and the dullness gradually to disappear, up to the present time, when there remains only a dullness in the lower part of the lung. The breathing in the left side is good throughout, except being somewhat puerile. The air and fluid appear to be absorbed, and the man is walking about the house. He sleeps well and has a good appetite, and has not had any bad symptom nor suffered any pain, except for four or five days after being shot.

---

Case 2.—May 22d, at 12 M., was called to see a young man nineteen years old, who had taken an ounce of laudanum, for the purpose of committing suicide. He had taken it at 11½ A. M. I found him in bed and asleep, but he was easily aroused. I had him taken up immediately, and after losing some time in persuading him to let me introduce the tube of my stomach pump, I pumped him out very thoroughly. But by this time an hour had elapsed since the taking of the laudanum, and sufficient impression had been made on the nervous system to produce a perfect prostration of the vital powers. He sank into a profound coma, his pulse scarcely perceptible, skin cold, face turgid and livid, with, at times, hardly a sign of life. Under these circumstances I thought I would try Marshall Hall's method of keeping up respiration, hoping, if I could do it for sufficient time, the brain might recover from the poison of the opium. These appalling effects took place an hour after taking the laudanum, and I kept up the motion prescribed by Dr. Hall, until 6 P. M., without any apparent amendment, when suddenly at that time he threw up his arms, and raised up in bed, and of course recovered. He remained excessively drowsy for three hours after, but I kept him awake by walking and flagellation. The only remarkable feature in this case, is the apparent success following the treatment by Dr. Hall's method of respiration in a case seemingly hopeless.

